# Measuring dissolution profiles of single controlled-release drug pellets

**DOI:** 10.1038/s41598-020-76089-z

**Published:** 2020-11-12

**Authors:** Heran C. Bhakta, Jessica M. Lin, William H. Grover

**Affiliations:** Department of Bioengineering, University of California, Riverside, Riverside, USA

**Keywords:** Biomedical engineering, Drug delivery, Pharmacology, Pharmaceutics, Drug delivery, Drug development, Techniques and instrumentation

## Abstract

Many solid-dose oral drug products are engineered to release their active ingredients into the body at a certain rate. Techniques for measuring the dissolution or degradation of a drug product in vitro play a crucial role in predicting how a drug product will perform in vivo. However, existing techniques are often labor-intensive, time-consuming, irreproducible, require specialized analytical equipment, and provide only “snapshots” of drug dissolution every few minutes. These limitations make it difficult for pharmaceutical companies to obtain full dissolution profiles for drug products in a variety of different conditions, as recommended by the US Food and Drug Administration. Additionally, for drug dosage forms containing multiple controlled-release pellets, particles, beads, granules, etc. in a single capsule or tablet, measurements of the dissolution of the entire multi-particle capsule or tablet are incapable of detecting pellet-to-pellet variations in controlled release behavior. In this work, we demonstrate a simple and fully-automated technique for obtaining dissolution profiles from single controlled-release pellets. We accomplished this by inverting the drug dissolution problem: instead of measuring the increase in the concentration of drug compounds in the solution during dissolution (as is commonly done), we monitor the decrease in the buoyant mass of the solid controlled-release pellet as it dissolves. We weigh single controlled-release pellets in fluid using a vibrating tube sensor, a piece of glass tubing bent into a tuning-fork shape and filled with any desired fluid. An electronic circuit keeps the glass tube vibrating at its resonance frequency, which is inversely proportional to the mass of the tube and its contents. When a pellet flows through the tube, the resonance frequency briefly changes by an amount that is inversely proportional to the buoyant mass of the pellet. By passing the pellet back-and-forth through the vibrating tube sensor, we can monitor its mass as it degrades or dissolves, with high temporal resolution (measurements every few seconds) and mass resolution (700 nanogram resolution). As a proof-of-concept, we used this technique to measure the single-pellet dissolution profiles of several commercial controlled-release proton pump inhibitors in simulated stomach and intestinal contents, as well as comparing name-brand and generic formulations of the same drug. In each case, vibrating tube sensor data revealed significantly different dissolution profiles for the different drugs, and in some cases our method also revealed differences between different pellets from the same drug product. By measuring any controlled-release pellets, particles, beads, or granules in any physiologically-relevant environment in a fully-automated fashion, this method can augment and potentially replace current dissolution tests and support product development and quality assurance in the pharmaceutical industry.

## Introduction

The rate at which an oral drug product releases its active ingredients into the body is profoundly important. In one particularly high-profile example, the painkiller OxyContin was developed by Purdue Pharma to provide the patient with a long-lasting “controlled release” of the active ingredient (the opioid oxycodone). By slowly releasing oxycodone from a matrix of water-insoluble materials over 12 h, OxyContin tablets are intended to require less frequent dosing than plain oxycodone. However, some patients taking OxyContin reported the return of pain and excruciating withdrawal symptoms before the end of their 12-hour doses, suggesting that the release of oxycodone from OxyContin was not as sustained as advertised^1^. To compensate, some doctors began prescribing additional opioids to “fill the gaps” in oxycodone delivery, and some patients sought relief with illicit painkillers, both of which led to increased potential for addiction. Ultimately, the inconsistent controlled release of OxyContin (and Purdue Pharma’s marketing of the product) was implicated as a major contributing factor to the ongoing opioid epidemic, which now kills over 130 Americans from overdoses every day^2^.

Developing controlled-release oral drug products is difficult in part because many factors can influence drug dissolution in the body. These factors include the pH and chemical composition of the gastrointestinal fluid, the hydrodynamics of the fluid caused by gastrointestinal motility, the patient’s metabolism and sex, and many other factors^3^. Indeed, something as simple as taking OxyContin with a high-fat meal can increase the amount of oxycodone in the patient’s blood by 25%^4^.

To predict how a solid drug product will dissolve in the body, pharmaceutical companies turn to in vitro dissolution testing. In these tests, the drug product of interest is placed in a vessel filled with fluid that mimics the contents of the gastrointestinal (GI) tract, the fluid or drug is stirred or agitated to recreate the hydrodynamics of the GI tract, and the concentration of drug in the media is measured periodically using a fraction collector and an analysis method like ultraviolet-visible spectroscopy (UV-VIS) or high-performance liquid chromatography (HPLC). The data from in vitro testing is used to create an in-vitro in-vivo correlation (IVIVC), a model used to predict a drug product’s *in vivo* performance based on its in vitro characteristics^5^.

Despite the ubiquity and importance of in vitro dissolution tests, the existing methods have significant shortcomings. For example, in the *USP II* method (which places the drug product on the bottom of the vessel with a spinning paddle stirring above it^6^), a cone-shaped zone of high concentration can form directly under the paddle which artificially decreases the dissolution rate of the drug product^7^. In the same test, having a tablet just 21 mm off-center in the vessel can increase the measured dissolution rate by a factor of two^8,9^. Also, in the *USP I* method (which places the drug product in a rotating wire basket^6^), the basket introduces other complications like clogging, impeded flow, and generation of air bubbles, all of which can affect the measured rate of dissolution^10^. Finally, the *USP IV* method addresses some of these shortcomings by pumping a continuous flow of fresh dissolution media past a drug product held in place by a filter^6^, but clogging within the filter can alter flow rates during the experiment, which again affects the measured dissolution rate^6,11^.

Additionally, the existing USP dissolution methods share several fundamental deficiencies: the measurement process is time-consuming, laborious, and often irreproducible. The actual dissolution of a single sample can take several minutes to several hours; added to this are the time and labor required for setup, fraction collection, and chemical analysis of the generated fractions. When combined with other sources of error, like variable calibration tablet quality, instrument problems, and poor operator training^12–14^, these factors make the existing USP dissolution methods highly irreproducible. Indeed, studies show that even when testing the dissolution of the exact same commercial drug product, different laboratories report significantly different dissolution rates despite using the same USP methods^15^.

Existing dissolution methods also provide only “snapshots” of the drug product dissolution process, making a measurement only whenever a fraction is collected. While these single-point measurements may be acceptable for some slow-releasing drug products, they provide limited information about how dissolution release rates may change over the dissolution process. Consequently, the US Food and Drug Administration (FDA) has recognized the deficiencies of single-point dissolution tests in their “Scale-Up and Post-Approval Changes” (SUPAC) guidance to drug makers^16,17^. SUPAC states that a drug dissolution *profile* (containing many measurements over time) can characterize a drug product more precisely than a single dissolution test, especially when studying the different behaviors of controlled-release drug products in different chemical and physical conditions. According to SUPAC, dissolution profile testing can not only help establish the efficacy and safety of a drug product, but also potentially reduce the need for in vivo testing, especially for minor drug reformulations and manufacturing changes. There is a need for new testing methods that can provide the complete drug product dissolution profiles recommended by SUPAC.

Finally, in many controlled-release medications, it is the capsule or tablet’s contents (not the capsule or tablet itself) that are designed to have controlled-release behavior. These controlled-release contents often take the form of many small pellets, particles, beads, granules, etc. inside each capsule or tablet^18^. When existing USP dissolution techniques are used to analyze whole multi-particle drug products like capsules and tablets, the measurements provide no information about the dissolution of individual pellets inside these products. Rather, the measured rate of dissolution is the combined rate of all of the pellets dissolving simultaneously. This measurement obscures any differences (either intentional or unintentional) in dissolution rates between the different pellets, information that could provide valuable insights into the consistency of the drug manufacturing process. And while it is possible to measure single-pellet dissolution rates using existing methods (e.g.,^19,20^), pellet floating and clumping make it difficult to use the common USP I and II methods with small numbers of pellets^20^, and these measurements still require sensitive detectors and suffer from poor temporal resolution (typically one measurement every 15 or 30 min).

In this work, we introduce a technique suitable for obtaining complete dissolution profiles from single controlled-release pellets, beads, and granules in an efficient and automated manner. We accomplished this by inverting the drug dissolution problem: instead of periodically measuring the *increase* in the concentration of drug compounds in the solution during dissolution (as is commonly done), we constantly measure the *decrease* in the microgram-scale mass of the solid pellet as it dissolves. This provides novel data that complements (and in some cases could replace) traditional dissolution measurements. Additionally, our mass-based dissolution testing requires no additional chemical analysis tools like UV-VIS or HPLC, is suitable for any drug product in any fluid environment regardless of their ingredients, is fully automated, provides much higher time resolution with measurements every few seconds, and can ultimately help accelerate the development of better controlled-release pharmaceuticals for better patient outcomes.

**Figure 1:**
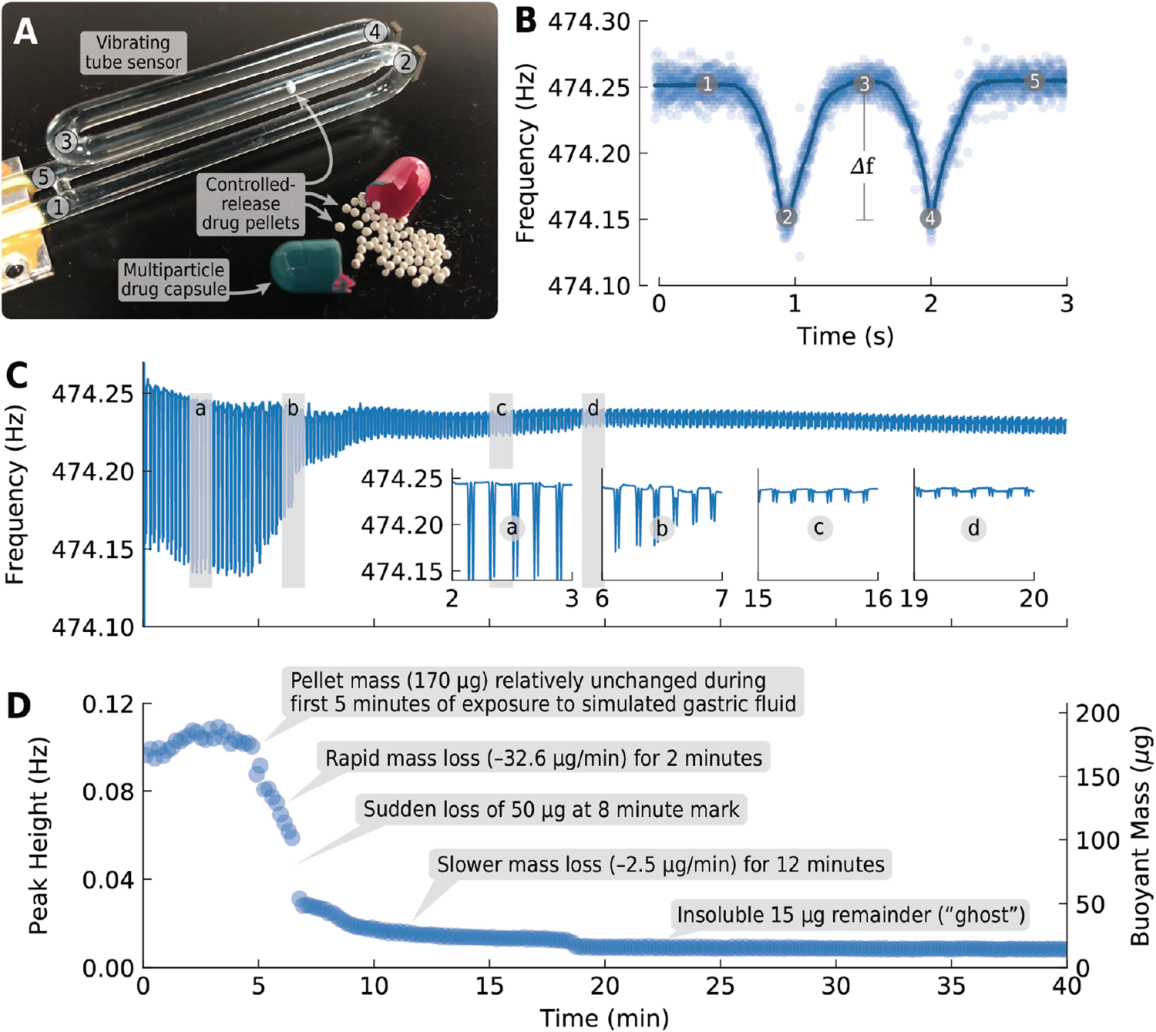
Using a vibrating tube sensor to obtain the dissolution profile of a single controlled-release pellet, bead, or granule obtained from a multi-particle drug product. The sensor **(A)** consists of a hollow glass tube bent in three places to form a tuning-fork shape and mounted so that the two “tines” of the fork (labeled 2 and 4) are free to move. The tube is filled with fluid, and an electronic feedback circuit (not shown) keeps the tube vibrating at its resonance frequency (474.25 Hz); this frequency is inversely proportional to the mass of the tube and its contents. When a pellet (in this case, a pellet from inside a capsule of the proton pump inhibitor lansoprazole) passes through the tube, the pellet causes the tube’s resonance frequency to change momentarily by an amount that is proportional to the buoyant mass of the pellet. This change is recorded as two peaks in the plot of resonance frequency vs. time **(B)**; the labels 1–5 on this plot correspond to the pellet’s position at points 1–5 in **(A)**, and the height of the peaks in **(B)** (about 100 mHz or 0.1 Hz) are proportional to the buoyant mass of the pellet (about 180 $\upmu$g). By repeatedly passing the pellet back and forth through the tube as the pellet dissolves and plotting the resonance frequency vs. time **(C)**, the shrinking peak heights record the dissolution of the pellet over the 40-minute experiment. The inset plots **(a)** through **(d)** provide closeup views of the peaks at 2, 6, 15, and 19 minutes. Finally, by plotting peak height vs. time and applying the tube’s calibration factor (**D)**, we can observe different pellet dissolution rates and other meaningful events throughout the dissolution process.

Our method uses vibrating glass tubes as simple but sensitive mass sensors. These sensors (Fig. 1A) consist of a length of glass tubing bent in three locations to form a tuning-fork-like shape. The glass is mounted at the bottom, leaving the “tines” of the glass tuning fork free to move and vibrate. Just like a traditional tuning fork, the glass tube vibrates predominantly at a single frequency, its resonance frequency, which is a function of the tube’s mass, shape, and other factors. Small magnets at the tips of the tube’s “tines” align with coils of wire, which are in turn connected to an electrical circuit that keeps the tube vibrating at its resonance frequency. When the tube is filled with fluid, the fluid’s mass causes the tube’s resonance frequency to drop by an amount proportional to the density of the fluid (in this manner, vibrating glass tubes have been used as fluid density sensors for nearly 50 years^21,22^).

When a controlled-release pellet flows through a fluid-filled vibrating tube sensor, the pellet momentarily replaces a volume of fluid equivalent to the pellet’s volume^23^. If the pellet’s density $\rho _{\mathrm {pellet}}$ is exactly equal to the fluid density $\rho _{\mathrm {fluid}}$, then the pellet’s presence in the sensor has no effect on the total mass of the sensor, so the resonance frequency of the sensor remains unchanged. However, if the pellet is more or less dense than the fluid (as is usually the case), then its presence adds or subtracts mass from the sensor. This causes the resonance frequency of the sensor to momentarily decrease (if $\rho _{\mathrm {pellet}} > \rho _{\mathrm {fluid}}$ ) or increase (if $\rho _{\mathrm {pellet}} < \rho _{\mathrm {fluid}}$ ). The magnitude of the frequency change is proportional to the pellet’s buoyant mass $m_{b}$:

1 $$\begin{aligned} m_{b} = m \left( 1 - \frac{\rho _{\mathrm {pellet}}}{\rho _{\mathrm {fluid}}} \right) \end{aligned}$$

where *m* is the absolute (in vacuo) mass of the pellet. By measuring the change in resonance frequency when a pellet passes through a vibrating tube and applying the tube’s calibration constants (obtained by measuring pellets of known masses in fluids of known densities), we can determine the buoyant mass of the pellet.

Figure 1B shows a plot of the resonance frequency of a vibrating tube sensor while a single pellet of a controlled-release drug product passes through the sensor. In this example, the pellet is from the interior of a capsule of the commercial proton pump inhibitor lansoprazole pictured in Fig. 1A, and the fluid filling the sensor is simulated gastric fluid (details below). The numbers 1 through 5 in Fig. 1B mark the resonance frequency of the sensor when the pellet is located at the same-numbered points inside the sensor in Fig. 1A. The pellet starts at point 1 at the base of the vibrating tube. Since this portion of the tube does not vibrate, the recorded resonance frequency of the tube is unchanged by the presence of the pellet (flat region at label 1 in Fig. 1B). But as the pellet moves into the first vibrating “tine” of the tube, the resonance frequency starts to change (decreasing, in this case, because the lansoprazole pellet is more dense than the simulated gastric fluid). When the pellet reaches the tip of the first tine (point 2 in Fig. 1A) where the vibrational amplitude is highest, its effect on the resonance frequency is greatest (corresponding to the tip of the downward peak labeled 2 in Fig. 1B). Then the pellet travels to midpoint between the two tines (point 3 in Fig. 1A), where the tube’s vibrational amplitude is again zero and its resonance frequency returns to baseline (labeled 3 in Fig. 1B). The pellet then travels to the tip of the second tine (point 4 in Fig. 1A) where the pellet’s mass changes the tube’s resonance frequency a second time, resulting in the peak labeled 4 in Fig. 1B. Finally, the pellet returns to the base of the vibrating tube (point 5 in Fig. 1A) and the resonance frequency of the tube again returns to baseline (labeled 5 in Fig. 1B). In this manner, the passage of a pellet through a vibrating tube sensor is recorded as two peaks in the plot of resonance frequency vs. time, with the heights of these peaks proportional to the buoyant mass of the pellet.

By repeatedly passing the same pellet back-and-forth through the sensor, we can continuously monitor the buoyant mass of the pellet and measure the rates at which the pellet is losing mass. Figure 1C shows sample data from measuring the buoyant mass of the same lansoprazole pellet hundreds of times over 40 min. Each pair of downward peaks corresponds to a single passage of the pellet through the sensor. The peaks get smaller as the pellet dissolves. By plotting peak height vs. time and converting the frequency change to buoyant mass using the sensor’s calibration data, we obtain the complete single-pellet dissolution profile as shown in Fig. 1D.

The dissolution profile of the lansoprazole-containing pellet in Fig. 1D shows that the pellet starts with a buoyant mass of about 170 $\upmu$g and remains relatively unchanged for its first 5 minutes in simulated gastric fluid. The pellet then begins losing mass at a rate of about –33 $\upmu$g/min for 2 min. At the 7-min mark, the pellet suddenly loses about 50 $\upmu$g—nearly half of its remaining mass—within 10 s. Now at about 50 $\upmu$g (or about a third of its starting mass), the pellet slowly loses about 30 $\upmu$g more mass over the next 12 min, a rate of about –2.5 $\upmu$g/min. By the 18-min mark, only about 15 $\upmu$g of the pellet remains, and its mass remains unchanged for the remaining 22 min. This insoluble remainder of a pellet is sometimes called a “ghost” when it is visible in a patient’s feces^24^. This pellet’s “ghost” represents only about 9% of the original starting mass of the pellet.

As the previous paragraph shows, dissolution profiles like the one in Fig. 1D can yield several different qualitative and quantitative metrics of the pellet’s size, composition, and behavior. Many of these metrics would have been difficult or impossible to obtain using the conventional USP methods (for example, the sudden loss of half of the pellet’s mass in 10 s at the 7-min mark). For pharmaceutical researchers developing controlled-release drug products, this data can provide valuable insights into the performance of their products. And by measuring single pellets, our technique provides novel information on pellet-to-pellet variation in dissolution behavior, information that provides insights into the consistency of the manufacturing process. Finally, this method is fully automated, needs no additional analysis techniques like UV-VIS or HPLC, and requires only as much time as it takes for a sample to dissolve.

## Results

To further validate our technique, we used vibrating tube sensors to obtain single-pellet dissolution profiles for three different over-the-counter commercial oral drug products: omeprazole (generic; Walgreen Company, Deerfield, IL), lansoprazole (generic; CVS Caremark Corporation, Woonsocket, RI), and esomeprazole (brand name Nexium; Pfizer, New York, NY). These drugs are all proton pump inhibitors intended to reduce stomach acid production, but they do not act from inside the stomach. Rather, an enteric coating on the pellets inside the capsules keeps the pellets intact inside the stomach and delays dissolution until the pellets enter the small intestine, where the less-acidic environment causes the enteric coating to break down and release the drug^25,26^. The drug is then absorbed through the intestines into the bloodstream, where it travels back to the stomach and inhibits acid production by blocking the proton pump system in gastric parietal cells.

**Figure 2:**
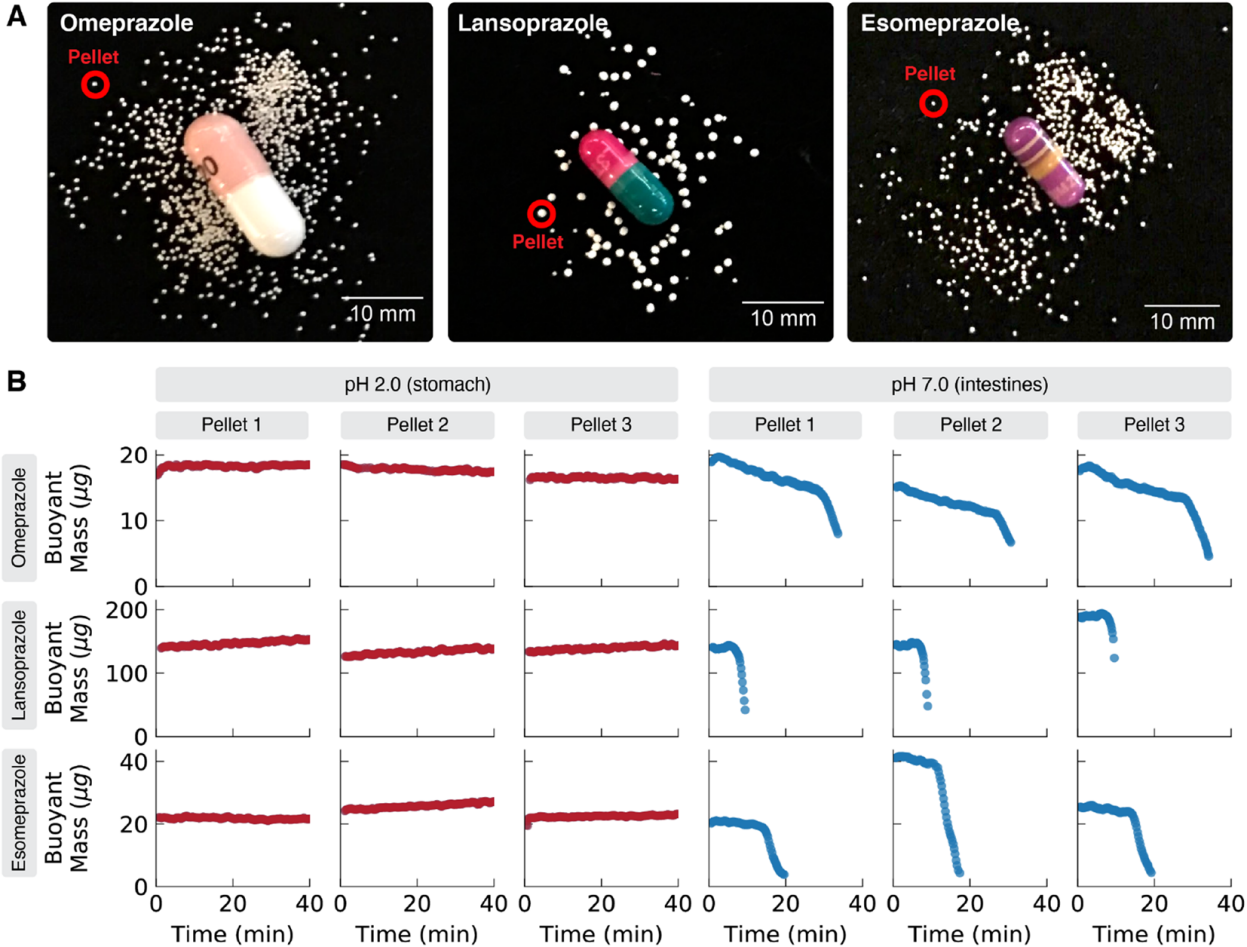
Using vibrating tube sensors to obtain single-pellet dissolution profiles **(B)** for three different over-the-counter proton pump inhibitor drugs, omeprazole, lansoprazole, and esomeprazole, in gastric fluids simulating the contents of the stomach (pH 2.0; red points) and the intestines (pH 7.0; blue points). For each drug, three separate controlled-release pellets (like the ones circled in red in **A**) were removed from capsules and tested at each pH value. In the simulated stomach contents at pH 2.0, the enteric coatings on the pellets protected the pellets from dissolution, and the measured pellet masses remain largely unchanged for at least 40 min. In contrast, in the simulated intestinal contents at pH 7.0, all three types of pellets dissolve within a few minutes, though they do so in very different ways. The omeprazole-containing pellets (generic; Walgreens Pharmacy) begin dissolving immediately, slowly losing mass at a rate of –0.18 $\upmu$g/min for about 30 min, then abruptly losing the remaining mass at a rate of –1.5 $\upmu$g/min and dissolving completely by the 35 min mark. In contrast, the lansoprazole-containing pellets (generic; CVS Pharmacy) remained unchanged for the first 10 min, then suddenly dissolved away in less than 2 min. Finally, the esomeprazole-containing pellets (brand name Nexium; Pfizer) initially dissolved slowly at a rate of –0.20 $\upmu$g/min for the first 15 min, then abruptly switched to a faster dissolution rate of –4.4 $\upmu$g/min and dissolved completely by the 20 min mark. The similarities between the different pellets from the same drug product suggest good pellet-to-pellet consistency in the manufacture of these controlled release formulations, and the differences between the different drugs indicate that the different products have different controlled release mechanisms (and consequently may have different dosing behavior) despite all having the same intended function in the body.

Our samples of omeprazole, lansoprazole, and esomeprazole all came from the manufacturers as multi-particle capsules, each of which contained several small delayed-release pellets, as shown in Fig. 2A. Since it is the pellets (not the capsules that contain them) that have controlled-release behavior, we manually opened and emptied the capsules, discarded the empty capsule shells, and used our technique to obtain single-pellet dissolution profiles for several controlled-release pellets from each capsule. The pellets ranged in sizes from about 400 $\upmu$m for omeprazole, 450 $\upmu$m for esomeprazole, and 900 $\upmu$m for lansoprazole.

Pellets from each drug product were tested in two different simulated gastric fluids: simulated stomach contents (pH 2.0) and simulated intestinal contents (pH 7.0); detailed ingredients in “Methods” below. After filling the vibrating tube sensor with a gastric fluid, a single pellet was added to the tube and a computer-controlled peristaltic pump was used to pass the pellet back and forth through the tube every 10 s for approximately 2 h or until the pellet had completely dissolved. This was repeated for several different pellets from each drug product.

The results in Fig. 2 show significant similarities and differences among both the three types of medications and the different pellets of each type of medicine. In simulated stomach contents (pH 2.0), the enteric coatings on all three types of pellets remained intact, and the masses of the pellets remain unchanged for at least 40 min of exposure to the stomach conditions (see below for longer-duration experiments in simulated stomach contents). However, in simulated intestinal contents (pH 7.0), the enteric coatings of all three types of pellets broke down as intended, and the masses of the pellets began to drop as their contents were released. Interestingly, the different drug products had very different single-pellet dissolution profiles in the simulated intestinal contents:

- *Omeprazole-containing pellets* in simulated intestinal fluid had the smallest starting mass of all the proton pump inhibitors at 15–20 $\upmu$g per pellet. The omeprazole-containing pellets also had the slowest dissolution rate, with each pellet requiring over 30 min to dissolve completely. Additionally, the omeprazole-containing pellets demonstrated a unique two-phase dissolution profile, losing mass at a relatively-slow rate of about –0.18 $\upmu$g/min for the first 30 min, then abruptly shifting to a faster rate of about –1.5 $\upmu$g/min for the remaining 5 min. This suggests that the omeprazole-containing pellets may have a more complex (possibly multi-layer) design that affects their controlled release behavior. The release rates were largely consistent across the different omeprazole-containing pellets.
- *Lansoprazole-containing pellets* in simulated intestinal fluid had the largest starting mass at 120–200 $\upmu$g per pellet (about ten times more massive than the omeprazole-containing pellets). The lansoprazole-containing pellets also had the fastest dissolution rates: the pellets lasted only about 10 min before suddenly dissolving away in less than 2 min. Additionally, even though different lansoprazole-containing pellets have significantly different starting masses, they all released their drug payloads at roughly the same time (after about 10 min in simulated intestinal fluid).
- *Esomeprazole-containing pellets* in simulated intestinal fluid had the largest variation in starting pellet masses, ranging from 20 to 40 $\upmu$g per pellet. But despite this variation, all of the esomeprazole-containing pellets yielded similar dissolution profiles, dissolving slowly at about –0.20 $\upmu$g for about 15 min before rapidly dissolving at a rate of about –4.4 $\upmu$g/min and disappearing completely by the 20 min mark.

These results again demonstrate the value of single-pellet dissolution profiles in characterizing controlled-release drugs. For example, by measuring single pellets, our method clearly resolves the two distinct release rates in the omeprazole-containing pellets and confirms that these rates are roughly constant across the different pellets; this information would be difficult or impossible to obtain using conventional USP dissolution methods that measure the average dissolution of the hundreds of pellets in a single capsule. Also, the high temporal resolution of our method (measuring pellet mass every 10 s) allows us to clearly resolve the very fast (< 2 min) dissolution of the lansoprazole-containing pellets; this detail would be lost in conventional techniques that collect analysis fractions every few minutes. For drug products with high variability in starting pellet size, like the esomeprazole-containing pellets, our technique can determine whether the different-sized pellets have different dissolution rates; this information would again be very difficult to obtain using existing techniques that usually measure ensembles of pellets. Finally, while all three drug products have the same intended function (proton pump inhibition), our results indicate that the pellets have different controlled release mechanisms, and these differences could lead to different patient treatment efficacies among the drug products.

Our results from Fig. 2 made us wonder, if there are measurable variations in single-pellet dissolution profiles across different drugs, are there similar variations between different formulations of the *same* drug? Specifically, are there differences in single-pellet dissolution behavior between name-brand and generic formulations of a drug? Generic formulations are frequently viewed as equivalent to and interchangeable with their name-brand versions; many states even have laws that *mandate* substitution of less-expensive generics for name-brand drugs when available, and the vast majority of prescriptions in the US are filled using generics^27^. The FDA requires manufacturers of generic controlled- or delayed-release drugs to demonstrate similar drug release behavior compared to the name-brand versions^28^, though there have been cases of generic versions of drugs that were less effective (or even dangerous) compared to their name-brand equivalents (e.g.,^29^). Our single-pellet dissolution profiles could help manufacturers and regulators identify differences between generic and name-brand dissolution rates in vitro before they cause adverse patient outcomes in vivo.

**Figure 3:**
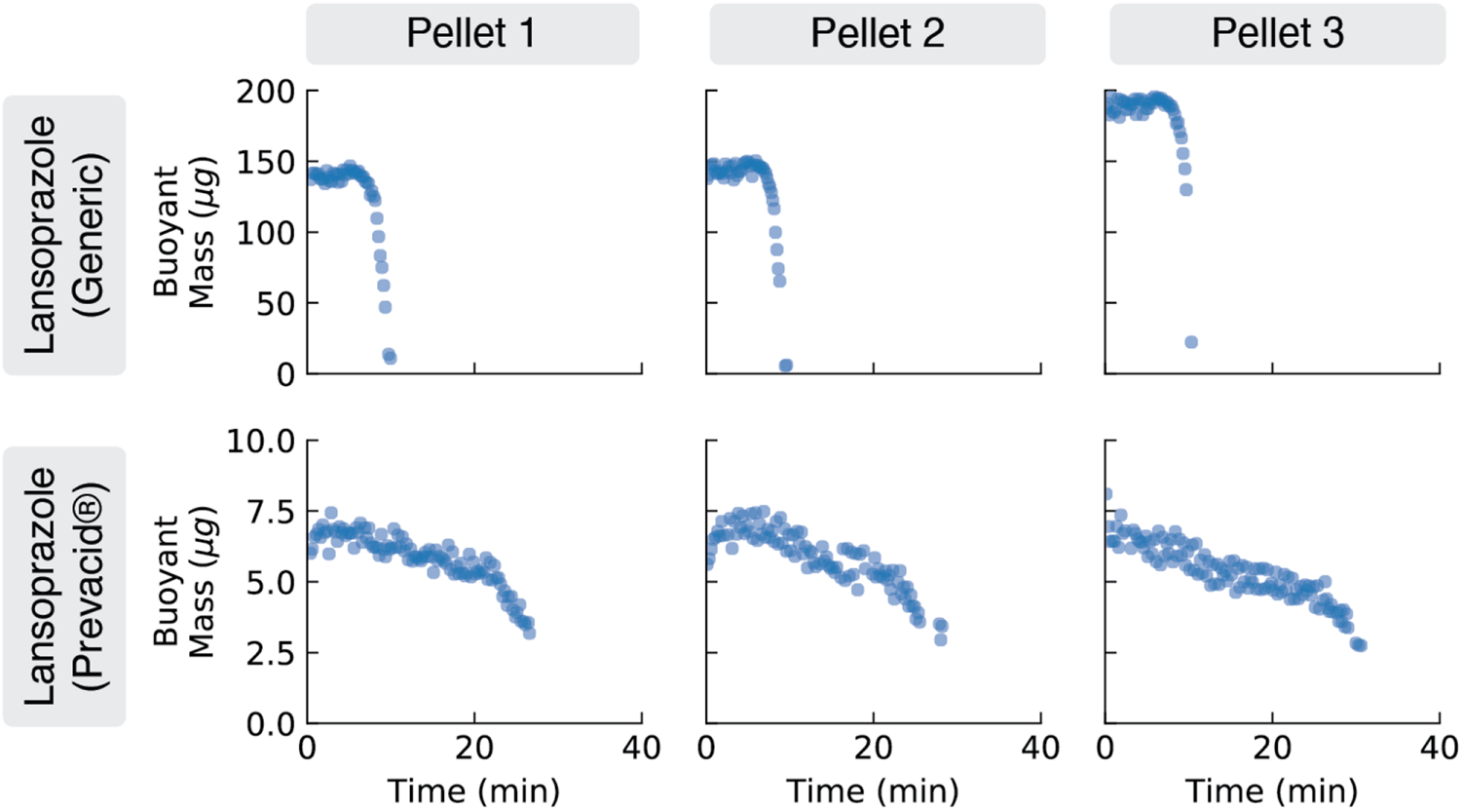
Using vibrating tube sensors to measure single-pellet dissolution profiles for two different controlled-release drug products containing the same dose of the same active ingredient, lansoprazole. For each drug product, three pellets were tested in simulated intestinal fluid at pH 7.0. The generic lansoprazole-containing pellets had the largest starting masses (150 to 200 $\upmu$g) and the fastest dissolution (disappearing completely in only 10 min). In contrast, the name-brand lansoprazole-containing pellets had the smallest starting masses (only around 7 $\upmu$g) and the slowest dissolution (dissolving at –100 ng/min for the first 20 min, then accelerating to a dissolution rate of –400 ng/min until the pellets fully dissolved at the 30 min mark. These results show that different formulations of the same active ingredient can have dramatically different single-pellet dissolution profiles, and may help explain any clinically-observed differences in the behaviors of these products.

Figure 3 compares dissolution profiles for two different drug products with the same active ingredient (lansoprazole), same dose of the active ingredient (15 mg), and same controlled-release time profile (24 h). The products differed only in their manufacturer and formulation: the generic product (CVS Caremark Corporation) consisted of large (900 $\upmu$m diameter) lansoprazole-containing pellets in a capsule, and the name-brand product (Prevacid; Takeda Pharmaceutical Company, Deerfield, IL) consisted of small (200 $\upmu$m diameter) lansoprazole-containing pellets in a quick-dissolving “SoluTab” tablet matrix.

The single-pellet dissolution profiles in Fig. 3 reveal significant differences between the generic and name-brand forms of this drug. While the generic lansoprazole-containing pellets are large (starting masses between 150 and 200 $\upmu$g) and dissolve away completely in only 10 minutes, the name-brand lansoprazole-containing pellets are much smaller (starting masses around 7 $\upmu$g) and dissolve more slowly over the course of about 30 min. Additionally, the name-brand lansoprazole-containing pellets appear to have a two-phase dissolution profile, with an initial slow dissolution rate of –100 ng/min for the first 25 minutes, followed by a faster average release rate of –400 ng/min for the next 5 min. On their own, these differences in single-pellet dissolution profiles do not prove that these two drug products will perform differently in patients’ bodies. However, this data could help explain clinically-observed differences. For example, based on this data, one might expect that levels of lansoprazole in the bloodstream might spike sooner after a patient takes the generic product with its fast-dissolving and larger pellets, while lansoprazole levels might rise more slowly in a patient that takes the name-brand product with its slow-dissolving and smaller pellets.

Finally, having used vibrating tube sensors to obtain single-pellet dissolution profiles over periods shorter than an hour, we wanted to assess our technique’s ability to monitor single-pellet masses over much longer time periods. This capability would be especially important for analyzing extended-release formulations that could take several hours to dissolve completely (like 12-h OxyContin).

**Figure 4:**
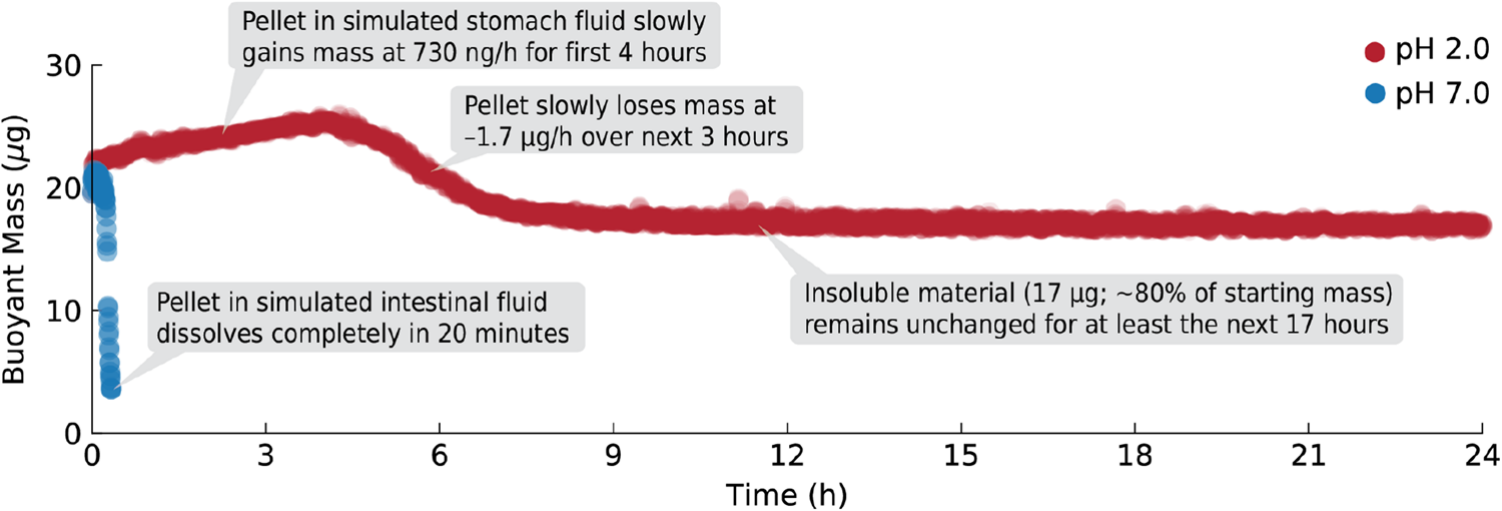
Measurements of the buoyant mass of a single controlled-release esomeprazole-containing pellet in simulated stomach contents (pH 2.0; red points) and simulated intestinal contents (pH 7.0; blue points) obtained every few seconds for 24 h. These results confirm that vibrating tube sensors can measure extremely slow dissolution rates (nanograms per hour) over extended periods of time.

Figure 4 shows 24-h-long single-pellet dissolution profiles for esomeprazole-containing pellets (brand name Nexium) obtained in both simulated stomach contents (pH 2.0; red points) and simulated intestinal contents (pH 7.0; blue points). As expected, in simulated intestinal contents the pellet dissolved completely in only 20 min; this result is consistent with our other measurements of proton pump inhibitors in pH 7.0 fluid. However, in simulated stomach contents, the pellet retained most of its mass during 24 h of exposure to the pH 2.0 fluid.

Closer inspection of the dissolution profile in Fig. 4 reveals additional details about the pellet’s dissolution behavior. During the first 4 h in simulated stomach fluid, the esomeprazole-containing pellet actually *gained* a small amount of mass at a rate of 730 ng/h, growing from 22 to 25 $\upmu$g (a 14% increase). We consider possible explanations for this small mass increase in “Discussion” below. Then, from 4 to 7 h, the pellet slowly *lost* mass at a rate of about $-1.7$ $\upmu$g/h. The total mass lost during hours 4 through 7 was greater than the mass gained from hours 0 through 4, indicating that some of the pellet’s contents are being released into the simulated stomach fluid during this time. However, after hour 7, the mass of the pellet remains unchanged for at least the next 17 hours. The final mass of the pellet, 18 $\upmu$g, is only about 20% less than the starting mass of the pellet and may correspond to the mass of a cellulose-based core that is known not to disintegrate^30^.

The full 24-h duration of the experiment in Fig. 4 has little basis in human physiology, since the contents of the stomach are typically completely emptied into the small intestine within 5 h of ingestion^31^. Indeed, since most of the observed pellet mass loss occurred after the 5-h mark, one could claim that the pellet’s enteric coating functioned as intended, remaining intact over the physiologically-relevant portion of the timeframe in Fig. 4. Regardless, these results show that vibrating tube sensors can weigh a single microgram-scale pellet for an entire day (obtaining nearly 10,000 mass measurements in the process) to measure extremely slow dissolution rates (on the order of nanograms per hour).

## Discussion

In this work, we show that a simple and inexpensive sensor can automatically measure the dissolution of single microgram-sized controlled-release pellets in physiologically relevant fluids with nanogram-scale resolution. This technique addresses many of the shortcomings of existing USP testing methods, requires no additional analytical instrumentation like UV-VIS or HPLC, and is suitable for both fast-dissolving and slow-dissolving formulations. And by obtaining dissolution profiles for single pellets instead of populations of pellets, our technique is capable of measuring pellet-to-pellet variations in dissolution behavior that are much more difficult to measure using existing methods. Using this technique, we observed significant variations in single-pellet dissolution profiles, not only between different types of drugs in different physiological conditions, but also between generic and name-brand formulations of the same drug, and even between different pellets from the exact same capsule.

This technique measures the buoyant mass of the pellet. As a measurement, buoyant mass has some advantages and disadvantages. One potential disadvantage is that the pellet’s density must be different than the density of the solution around it for our technique to measure the pellet. However, in the unlikely event that a pellet’s density equals the solution density, one can simply add inert substances to the fluid to change its density (like colloidal silica to increase the fluid density^32^) and render the pellet measurable. Additionally, pellet buoyant mass is sensitive to changes in both pellet mass and pellet density. For example, the small increase in the buoyant mass of a pellet containing esomeprazole (Nexium) that we measured in Fig. 4 could have been caused by high-density ingredients from the simulated gastric fluid entering the pellet, or low-density components of the pellet leaving the pellet. In practice, a typical user with a priori knowledge of the composition and construction of the pellet could probably determine which of these scenarios was most likely. And in cases where they cannot, a user can measure a pellet’s buoyant mass in two fluids of different densities (either using the same sensor^32^ or two sensors in series^33^) and calculate the mass, volume, and density of the pellet to determine which of these physical properties are responsible for the observed changes during pellet dissolution. In this manner, measuring a pellet’s buoyant mass during dissolution provides many useful insights into the chemical and physical changes occurring as the pellet dissolves.

Additionally, our technique is sensitive to both the mass of the active ingredient(s) and the mass of all the other components of a pellet. This is again both an advantage and a disadvantage. For applications that require specific chemical information about the active ingredient during dissolution (for example, the rate at which the concentration of the drug is increasing in the surrounding fluid), vibrating tube sensors may not be able to provide this specific information by themselves, and other techniques like UV-VIS and HPLC may still be necessary. In that case, by using a fraction collector to sample from the fluid contents of the vibrating tube sensor during operation and subjecting these fractions to traditional chemical analysis, one could obtain a complete picture of pellet dissolution that captures changes in both the pellet and the surrounding fluid. In other scenarios where the active ingredient (or a homogenous matrix containing the active ingredient) represents the majority of the mass of the pellet, then the loss of pellet mass measured by our technique might be a direct replacement for traditional measurements of active ingredient concentration in the surrounding fluid, thereby eliminating the need for additional techniques like UV-VIS and HPLC. Finally, if particles of a pure chemical compound are used, our technique could be used to measure the solubility product constant ($K_{sp}$) and other physiochemical properties of the compound in any desired fluid; this might be especially useful in early phases of drug development when only small amounts of a compound are available for characterization.

This technique provides pharmaceutical researchers and producers with a simple, low-cost, and fully automated tool for obtaining single-pellet dissolution profiles from *any* drug in *any* desired fluid. This capability should be powerful in a variety of different scenarios. For example, measurements of the dissolution behavior of pellets from each production batch can provide valuable quality assurance data and illuminate possible production defects before the product reaches consumers. Even within a single batch, single pellet dissolution profiles provide information about the consistency of the pellet manufacturing process. And as a gravimetric (mass-based) method, this technique places no constraints on the chemical or physical composition of the fluid surrounding the pellet, meaning that pharmaceutical developers are free to measure pellet dissolution in any physiologically-relevant fluid without fear that the fluid will interfere with the measurement process. For these reasons, vibrating tube sensors should help facilitate the development of better controlled-release pharmaceuticals with better patient outcomes.

## Methods

Vibrating glass tube sensors can be made by hand using a flame to bend glass tubing into the desired shape^23^ or harvested from commercial fluid density meters. In this work we used a sensor from a DMA 35 fluid density meter (Anton-Paar; Graz, Austria) with the circuitry modified to provide access to the signal that drives the vibrating tube sensor at its resonance frequency. We connected this signal to a frequency counter input on a multifunction data acquisition device (PCI-6259; National Instruments, Austin, TX) and used a custom LabVIEW program to record the frequency of the signal (and therefore the resonance frequency of the vibrating glass tube) once for every period of the signal (roughly every 2 ms). A computer-controlled peristaltic pump (Cole-Parmer, Vernon Hills, IL) was used to pump simulated gastric fluid (details below) containing the pellet to be measured back and forth through the vibrating glass tube sensor. A second custom LabVIEW program reversed the direction of fluid flow through the sensor every 10 s. A custom Python program read the recorded resonance frequency measurements from the sensor, applied a digital low-pass filter, and finally identified the pairs of peaks corresponding to transits of the pellet through the sensor. For each peak pair, the software calculated the average height of the peaks (the difference between the tips of the peaks and the baseline, in Hertz) and multiplied this peak hight by the sensor’s point-mass calibration constant (described below) to obtain the buoyant mass of the pellet.

The sensor was calibrated for both bulk fluid density and point mass:

- For the bulk fluid density calibration, the sensor was filled with NaCl solutions with precisely known densities ranging from 1.00 to 1.08 g/mL^34^ and the sensor’s resonance frequency was recorded for approximately 10 min. The average resonance frequency was plotted against the known fluid density, and the slope [units of Hz per (g/mL)] was used as a calibration constant to convert sensor frequency measurements to fluid densities (see Supplemental Figure S1).
- For the point mass calibration, a microbead of known mass and density was passed through the sensor several times in a fluid of known density (water). Each passage of the bead through the sensor results in a momentary change in the sensor’s resonance frequency as shown in Fig. 1B. By measuring the average amount of frequency change $\Delta f$ for the bead and applying Eq. (1), we obtain the sensor’s point mass calibration constant (units of g per Hz) and use this constant to convert controlled-release pellet measurements from frequency changes to buoyant masses. Additionally, the width of the distribution of frequency change measurements was used to calculate the point mass resolution of the sensor, 700 ng (see Supplemental Figure S2).

Controlled-release pellets removed from multi-particle drug capsules were measured in simulated gastric fluids based on Clavel et al.^35^. The simulated intestinal fluid contained 4.8 g/L NaCl, 1.56 g/L NaHCO$_3$, 2.2 g/L KCl, and 0.22 g/L CaCl$_2$; pH 7.0. The simulated stomach fluid was prepared using the same recipe but with 0.5 M HCl added to lower the pH to 2.0.

## Supplementary information

Supplementary Information.
