# Supplementary material for: Measuring dissolution profiles of single controlled-release drug pellets
Source: Sci Rep. 2020 Nov 12;10:19734. doi: 10.1038/s41598-020-76089-z (PMC7661542; doi:10.1038/s41598-020-76089-z)
Supplement: Supplementary file 1 — Supplementary Information. [file 41598_2020_76089_MOESM1_ESM.pdf]

Online supplementary information: Measuring dissolution profiles of single controlled-release drug pellets

Heran C. Bhakta, Jessica M. Lin, and William H. Grover*

Department of Bioengineering, University of California, Riverside.

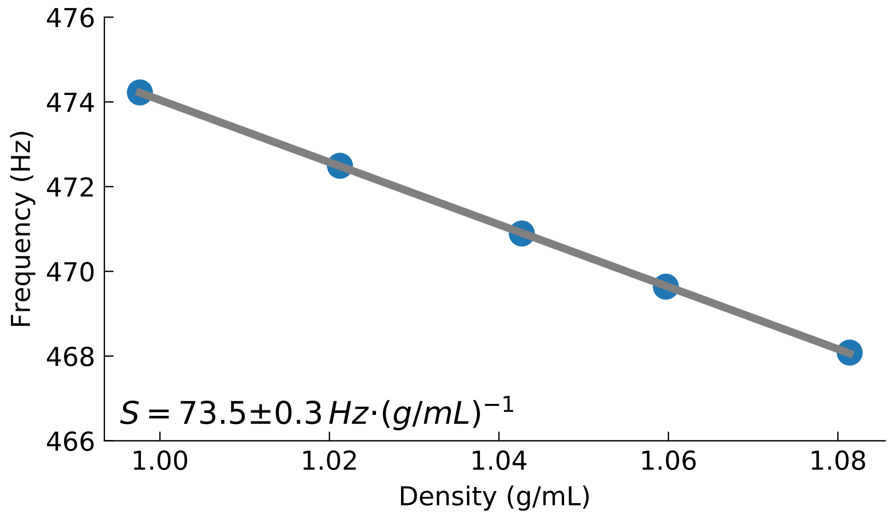

Figure S1: Bulk fluid density calibration of a glass vibrating tube sensor. Sodium chloride solutions with densities ranging from 1.00 to 1.08 g/mL were loaded into the sensor and the sensor's resonance frequency was recorded for 10 minutes; the average of these frequency measurements is plotted here versus the density of the fluid in the sensor during those measurements. The slope of this line, -73.5 ± 0.3 Hz $(\text{g/mL})^{-1}$, is used as a bulk fluid density calibration constant for the sensor.

*219 MSE, 900 University Ave., Riverside, CA 92521; wgrover@engr.ucr.edu

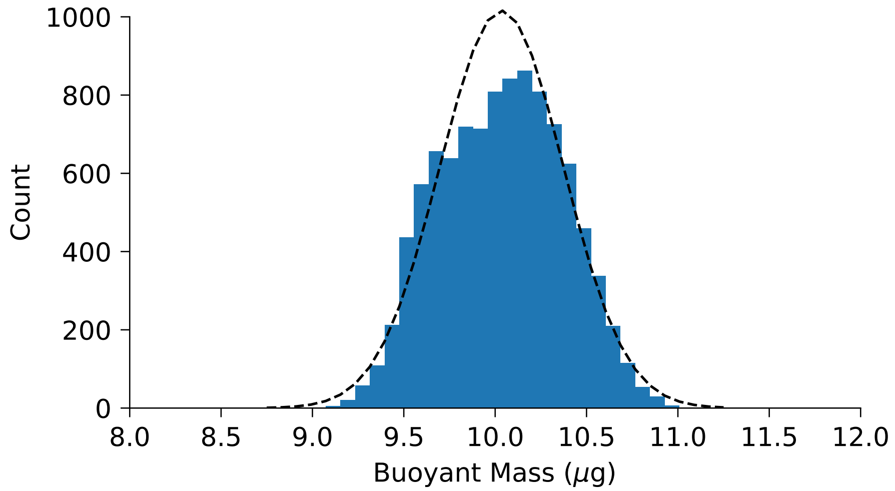

Figure S2: Point mass calibration of a glass vibrating tube sensor. A polyethylene bead of known mass and density (Cospheric; Santa Barbara, CA) was passed through a vibrating glass tube sensor in fluid 10,033 times. Dividing the average frequency change by the bead's known buoyant mass provides the point mass calibration constant for the sensor. The width of this distribution is also used to estimate the buoyant mass resolution of our this sensor, $\pm$678 ng.
